# Medical students and ChatGPT: analyzing attitudes, practices, and academic perceptions

**DOI:** 10.1186/s12909-025-06731-9

**Published:** 2025-02-05

**Authors:** Ahmed Samir Abdelhafiz, Maysa I. Farghly, Eman Anwar Sultan, Moaz Elsayed Abouelmagd, Youssef Ashmawy, Eman Hany Elsebaie

**Affiliations:** 1 Department of Clinical Pathology, National Cancer Institute, Cairo University, Kasr Al-Aini Street, Fom Elkhalig Square, Cairo, 11796 Egypt; 2 Department of Clinical and Chemical Pathology, Faculty of Medicine, Suez University, Suez, Egypt; 3 Department of Community Medicine, Faculty of Medicine, Alexandria University, Alexandria, Egypt; 4 Faculty of Medicine, Cairo University, Cairo, Egypt; 5 Department of Public Health and Community Medicine, Faculty of Medicine, Cairo University, Cairo, Egypt

**Keywords:** ChatGPT, Medical students, AI, Knowledge, Perceptions, Practices

## Abstract

**Background:**

ChatGPT, a chatbot launched by OpenAI in November 2022, has generated both excitement and concern within the healthcare education, research, and practice communities. This study aimed to explore the knowledge, perceptions, attitudes, and practices of undergraduate medical students regarding the use of ChatGPT and similar chatbots in their academic work.

**Methods:**

An anonymous, structured questionnaire was developed using Google Forms and administered to medical students as part of a cross-sectional study. The survey targeted undergraduate medical students from four governorates in Egypt. The questionnaire link was distributed through social media platforms, including Facebook and WhatsApp. The survey comprised four sections: socio-demographic characteristics, perceptions, attitudes, and practices.

**Results:**

The survey achieved a response rate of 96%, with 614 out of 640 approached students participating. Prior to the study, most respondents (78.5%) had personal experience using it. Overall, respondents demonstrated positive perceptions, attitudes, and practices toward ChatGPT, with mean scores exceeding 3 for all three variables: 3.99 ± 0.60 for perceptions, 3.01 ± 0.46 for attitudes, and 3.55 ± 0.55 for practices. In general, the students exhibited a high degree of trust in the model, with approximately half trusting the accuracy and reliability of the information provided by ChatGPT. However, more than two-thirds expressed apprehension about its potential misuse in medical education, and around 60% were concerned about the accuracy of information ChatGPT might generate on complex medical topics.

**Conclusions:**

Medical students show strong interest and trust in using ChatGPT and similar chatbots for academic purposes but have concerns about the reliability of the information and potential misuse in medical education. The use of AI tools should follow ethical guidelines set by academic institutions, with regular updates to keep pace with technological progress. Future research should focus on the impact of AI on education and personal development, especially among young people.

**Supplementary Information:**

The online version contains supplementary material available at 10.1186/s12909-025-06731-9.

## Background

Artificial Intelligence (AI) is the term used to describe the creation of computer systems that can carry out complex tasks which typically require human intelligence, such as making decisions, comprehending speech and identifying patterns or images [1].

The development of large language models and natural language processing, which seek to enable the automatic analysis of language, is one of the most notable advances in artificial intelligence. One of these applications is ChatGPT which was developed based on large language models. A more dynamic and interesting user experience is produced by this recently created AI technology, which allows users to participate in interactive conversations and receive human-like responses [2].

ChatGPT is a chatbot released by OpenAI in November 2022. It uses an enormous amount of data and advanced deep learning techniques to produce responses to user input that mimic those of a human as it can converse, respond to further inquiries, identify mistakes, and decline requests that are not appropriate [3].

ChatGPT has sparked both excitement and concerns in the context of health care education, research, and practice since its initial launch [4–6]. Quick and easy to use, ChatGPT has emerged as a valuable tool for medical instructors, patients, students, and healthcare providers [7–9].

Although some studies showed that utilization of ChatGPT is still in its early phases among medical researchers, a growing number of them were expressing interest in using it to improve their work as knowledge of its potential advantages grows [10].

Medical students can benefit from individualized learning experiences in a secure environment that can be modified to their requirements and learning styles using ChatGPT, which also offers instant feedback. Also, ChatGPT can be used as an assessment tool, giving students reasoned justifications for their wrong answers and enabling them to learn [11]. Furthermore, evidence-based information is available to students and trainees for use in academic writing, clinical care, and decision-making. This could translate into a better learning experience and, eventually, improved patient care [12, 13].

A systematic review of ChatGPT’s usefulness in health education was conducted recently [14]. Enhancing individualized learning experiences, possible improvement of communication skills, and raising student engagement in the learning process were among its mentioned benefits [8, 14–16].

On the other hand, several worries were brought up, such as the potential for producing false information and ethical concerns including the potential for plagiarism, bias and copyright violations [3, 14, 15].

Understanding the expectations, attitude, and perception of medical students is crucial as technological integration into medical education advances. This knowledge can help educators and policymakers to create and carry out successful educational interventions that are suited to the requirements of individual students [17].

Therefore, we aimed here to investigate the knowledge, perception, attitude and practice of undergraduate medical students in using ChatGPT and similar chatbots in their academic work.

## Methods

### Study design

This research was a cross-sectional study conducted among a sample of undergraduate medical students in Egypt, aiming to evaluate their knowledge, attitudes, and practices regarding the use of ChatGPT and similar chatbots in their academic work. The online survey was conducted in accordance with the guidelines of the Checklist for Reporting Results of Internet E-surveys (CHERRIES) [18] and complied with the reporting standards for observational studies outlined in the Strengthening of Observational Studies in Epidemiology (STROBE) Statement [19].

### Sample size and technique

The investigators employed a non-probability convenience sampling method known as “self-selection web-based questionnaires.” The questionnaire link was shared within community engagement groups on social media platforms (such as Facebook and WhatsApp) from May 1, 2024, to September 1, 2024. The survey was available in English. Along with the link, the investigators provided a statement explaining the purpose of the survey and included contact information for one of the researchers. The questionnaire was accessible to medical student groups in four selected governorates (Cairo, Alexandria, Suez, and Beni Suef) through the investigators’ academic connections, ensuring representation from different regions of Egypt. The inclusion criteria for participants were any undergraduate medical student in Egypt with access to electronic media and willing to participate. Exclusion criteria included individuals who were not affiliated with medical universities, lacked internet access, or chose not to participate.

The required sample size was calculated using the formula provided by OpenEpi:

Sample size n = [DEFF*Np (1-p)]/ [(d2/Z21-α/2*(N-1) + p*(1-p)].

Assuming 80% power, a 95% confidence level, a 5% significance level, and an estimated proportion of students expressing a positive attitude or trust in the reliability of ChatGPT at 40% [20], along with a design effect of 1, the minimum required sample size for this study was calculated to be 369 participants. To account for potential non-responses, an additional 40% (*n* = 148) was added, resulting in a total sample size of 517 participants.

### Data collection tool

The online data collection method involved creating a Google Forms survey, which was shared with participants for completion and submission. We reached out to students from four universities in four governorates, representing different regions of Egypt, through investigators affiliated with these universities.

The survey consisted of four sections:

1. Socio-demographic characteristics, previous knowledge, and usage of ChatGPT: This section gathered information on age, sex, governorate, type of university, as well as participants’ previous knowledge and usage of ChatGPT.
2. Perceptions: This section included three questions regarding the helpfulness of ChatGPT in understanding medical concepts, the accuracy of ChatGPT, and its usefulness in summarizing research papers. A scale ranging from 5 (Extremely helpful) to 1 (Not at all helpful) was used for responses.
3. Attitude: This section contained 18 questions designed with a 5-point Likert scale, from 5 (Strongly agree) to 1 (Strongly disagree). Topics covered included concerns about the reliability of ChatGPT’s information, the potential over-reliance on ChatGPT, and its impact on the development of critical thinking skills.
4. Practices: This section included 13 questions, also using a 5-point Likert scale (5: Strongly agree to 1: Strongly disagree). The questions focused on practical aspects such as the usefulness of ChatGPT for saving time when searching for information and recommending ChatGPT to colleagues to facilitate their academic tasks.

This anonymous structured questionnaire, used for medical students, was adopted from a similar study conducted in Jordan [21] (Supplementary Table 1).

### Pilot test

A subset of 52 participants (10% of the total sample size) was selected to evaluate and test the survey’s data collection instrument. This pilot test assessed the instrument’s suitability in terms of language, question style, and completion time. No changes were made following this test.

### Data analysis

The questionnaire was reviewed for completeness and logical consistency. Pre-coded data was entered into Microsoft Office Excel for Windows, 2016. Data was then double checked and transferred to Statistical Package for the Social Sciences, version 24 (SPSSV24). Graphs were generated using Microsoft Office Excel. All statistical analyses were performed using two-tailed tests and an alpha error of 0.05. A P-value ≤ 0.05 was considered statistically significant. Simple descriptive statistics (arithmetic mean and standard deviation) were used to summarize the quantitative data, and frequencies were used to summarize the qualitative data. An independent sample T-test was used to compare the perception, attitude and practices’ scores with demographic data. The mean score for each section was calculated as regards the 5-point scale (perception 3 questions, attitude 18 questions, practice 13 questions), and the arithmetic mean, and standard deviation were used for summarization. Percent scores for the three sections were calculated and categorized into two levels based on the mean. Scores below the mean were considered “fair,” while scores above the mean were considered “good.”

### Ethical considerations

Approval of the Institutional Review Board (IRB) at Faculty of Medicine, Alexandria University was obtained before starting the study (IRB No: 00012098). Objectives of the study were explained to the participants (by a statement before initiation of the online survey). Participants who refused were excluded from the study results and online consent was taken from those who accepted to participate. Strict confidentiality about participants’ personal data (this is secured by the questionnaire being anonymous) was maintained throughout data collection, entry and analysis (according to the Helsinki declaration).

## Results

This study was carried out on 614 subjects from four governorates in Egypt: Cairo, Alexandria, Suez and Beni-Suef to represent the different regions. The response rate was 96% (out of 640 students approached, 614 participated in the survey). Questionnaires were answered by all subjects on an online form in the period from May 2024 to September 2024. Most respondents (584, 95.1%) had heard of ChatGPT prior to the study, and 482 (78.5%) had used it.

The mean age of the students was 21 ± 2 years. Based on this, age was categorized into two groups: below the mean and above the mean. The sample was relatively balanced in terms of gender, with 314 males (51.1%) and 300 females (48.9%). Most participants were from Cairo (237, 38.6%), followed by Alexandria (176, 28.7%). Regarding university type, 522 respondents (85.0%) attended public universities, while 92 (15.0%) attended private universities. The demographic information of the respondents and information about their basic knowledge about ChatGPT are presented in Table 1.

**Table 1:** The demographic information of the respondents and their basic knowledge about ChatGPT

		*N*	%
**Age Categories**	**≤ 21 years**	348	56.7%
	**> 21 years**	266	43.3%
**Sex**	**Male**	314	51.1%
	**Female**	300	48.9%
**Governorate**	**Cairo**	237	38.6%
	**Alexandria**	176	28.7%
	**Suez**	135	22.0%
	**Beni-Suef**	66	10.7%
**University**	**Public**	522	85.0%
	**Private**	92	15.0%
**Have you heard of ChatGPT before this study?**	**Yes**	584	95.1%
	**No**	30	4.9%
**Have you used ChatGPT before this study?**	**Yes**	482	78.5%
	**No**	132	21.5%

Overall, the respondents reported positive perceptions, attitudes, and practices toward ChatGPT. The mean scores for all three variables were above 3, indicating a favorable overall assessment. However, there was some variation in scores, as evidenced by the standard deviations. For example, the perception scores had a slightly higher standard deviation compared to the attitude and practice scores, suggesting a wider range of opinions on this construct (Table 2).

**Table 2:** Perception, attitude, and practice scores towards ChatGPT

	Mean ± SD
**Perception score**	3.99 ± 0.60
**Attitude score**	3.01 ± 0.46
**Practice score**	3.55 ± 0.55

Table 3 presents the responses to some specific questions. Regarding students’ perception of ChatGPT’s accuracy in providing medical information, 64% found it helpful, 29.5% were neutral, and 6.5% thought it was not helpful. Regarding attitudes, some students voiced concerns about ChatGPT’s potential impact on academic skills and integrity. A large majority (67.8%) disagreed with the idea of over-relying on ChatGPT at the expense of critical thinking skills, and 62.9% were not worried about the reliability of the information it provided. However, there were concerns regarding security risks (19.9% agreed) and privacy issues (21.5% agreed). Additionally, many students feared that ChatGPT could compromise originality in assignments (21.5% agreed) or violate academic policies (27.0% agreed). Despite these concerns, 71.8% of students expressed interest in using ChatGPT to assist with their studies, while 71.3% were apprehensive about its potential misuse in medical education.

**Table 3:** Perception, attitude, and practice responses towards ChatGPT

Category	Question	Response	*N*	%
Perception	How likely do you think it is that ChatGPT provides accurate medical information?	Not helpful	40	6.5%
		Neutral	181	29.5%
		Helpful	393	64.0%
Attitude	I am concerned about the reliability of the information provided by ChatGPT	Disagree	386	62.9%
		Neutral	178	29.0%
		Agree	50	8.1%
	I am afraid of relying too much on ChatGPT and not developing my critical thinking skills	Disagree	416	67.8%
		Neutral	108	17.6%
		Agree	90	14.7%
	I am concerned about the potential security risks of using ChatGPT	Disagree	338	55.0%
		Neutral	154	25.1%
		Agree	122	19.9%
	I am afraid of becoming too dependent on technology like ChatGPT	Disagree	385	62.7%
		Neutral	126	20.5%
		Agree	103	16.8%
	I am afraid that using ChatGPT would result in a lack of originality in my university assignments and duties	Disagree	362	59.0%
		Neutral	120	19.5%
		Agree	132	21.5%
	I am afraid that the use of ChatGPT would be a violation of academic and university policies	Disagree	282	45.9%
		Neutral	166	27.0%
		Agree	166	27.0%
	I am concerned about the potential privacy risks that might be associated with using ChatGPT	Disagree	328	53.4%
		Neutral	154	25.1%
		Agree	132	21.5%
	I consider using ChatGPT for help with my studies	Disagree	41	6.7%
		Neutral	132	21.5%
		Agree	441	71.8%
	I am concerned about the potential for misuse of ChatGPT in medical education	Disagree	36	5.9%
		Neutral	140	22.8%
		Agree	438	71.3%
	I am concerned with the accuracy of information ChatGPT might generate on complex medical topics	Disagree	84	13.7%
		Neutral	167	27.2%
		Agree	363	59.1%
	I think ChatGPT could be a reliable source for preparing medical exams	Disagree	199	32.4%
		Neutral	188	30.6%
		Agree	227	37.0%
Practice	For me, ChatGPT is a reliable source of accurate information.	Disagree	101	16.4%
		Neutral	217	35.3%
		Agree	296	48.2%
	I think that relying on technology like ChatGPT can disrupt my critical thinking skills.	Disagree	360	58.6%
		Neutral	144	23.5%
		Agree	110	17.9%
	I appreciate the accuracy and reliability of the information provided by ChatGPT.	Disagree	84	13.7%
		Neutral	239	38.9%
		Agree	291	47.4%
	I believe that using ChatGPT can save time and effort in my university assignments and duties.	Disagree	27	4.4%
		Neutral	95	15.5%
		Agree	492	80.1%

As shown in Table 4, there were no significant differences in perception or attitude scores between the two age groups (p values of 0.775 and 0.811, respectively). The younger group (≤ 21 years) reported slightly higher practice scores compared to the older group (> 21 years). However, this difference was statistically insignificant (p value of 0.077). Regarding sex, there were no significant differences in perception or attitude scores between males and females (p values of 0.304 and 0.186, respectively). However, there was a significant difference in practice scores (p value of 0.014) where males reported slightly higher practice scores compared to females. Regarding university type, there were no significant differences in perception or attitude scores between public and private university students (p values of 0.411 and 0.234, respectively). However, there was a significant difference in practice scores (p value of 0.013) where private university students reported slightly higher practice scores compared to public university students.

**Table 4:** Comparison of perception, attitude, and practice scores among students based on age, gender, and university type

Category		Group	Mean ± SD	*P* value
Perception score	Age	≤ 21 years	3.99 ± 0.59	0.78
		> 21 years	3.97 ± 0.61	
	Gender	Male	4.09 ± 0.64	0.304
		Female	3.96 ± 0.54	
	University type	Public	3.97 ± 0.59	0.41
		Private	4.03 ± 0.60	
Attitude score	Age	≤ 21 years	3.01 ± 0.46	0.81
		> 21 years	3.04 ± 0.46	
	Gender	Male	3.03 ± 0.46	0.186
		Female	2.98 ± 0.44	
	University type	Public	3.01 ± 0.46	0.23
		Private	2.95 ± 0.42	
Practice score	Age	≤ 21 years	3.58 ± 0.53	0.08
		> 21 years	3.50 ± 0.57	
	Gender	Male	3.60 ± 0.54	0.014
		Female	3.49 ± 0.56	
	University type	Public	3.52 ± 0.54	0.01
		Private	3.68 ± 0.56	

## Discussion

While chatbots have been utilized for various purposes for years, ChatGPT has garnered significant attention since its release due to its open-access availability and remarkable ability to generate human-like text, write essays, translate and summarize lengthy texts, and answer complex questions [22]. These capabilities have raised the interest of the academic community, leading to extensive use of chatbots in teaching, education, and research [10, 23]. Our findings demonstrate a high level of awareness and usage of ChatGPT among medical students, reflecting the chatbot’s popularity within this group.

Previous studies have explored the positive attitudes of students towards chatbots [21, 24]. This is not surprising, as younger generations tend to show interest in new technologies more than older individuals. Notably, approximately three-quarters of our study participants reported using ChatGPT, which is significantly higher than the percentage of researchers who used the chatbot for academic purposes in our previous study [10].

While age did not influence chatbot usage practices in our group, gender was a significant factor, with male students exhibiting significantly higher practice scores than females. Research has shown that men tend to outpace women in generative AI usage across all age groups, with the gap being widest among younger generations [25–27]. This disparity may be attributed to cultural or societal factors that encourage males to engage more readily with technology and explore new tech tools.

Affiliation also emerged as a significant factor, with students from private universities showing significantly higher practice scores compared to those from public universities. Private institutions may offer more resources, focused institutional goals, and better access to advanced technologies, including paid AI models, which could explain the higher practice scores among their students. This difference could also reflect disparities in available infrastructure and attitudes toward technology between private and public institutions.

Most students in our study did not express concerns regarding the reliability of information provided by ChatGPT, while approximately one-fifth highlighted security and privacy risks associated with its use. A review of 24 studies on students’ perceptions and use of AI chatbots in higher education underscores a global interest in how students engage with these tools. In line with our findings, the review indicated that students find AI chatbots highly valuable and motivating, particularly for tasks such as personal assistance, immediate feedback, and support with writing, coding, and academic activities. However, the review highlighted persistent concerns about the accuracy and reliability of chatbot responses, as well as potential negative impacts on learning processes, critical thinking, discipline, and creativity [28].

While the use of chatbot technology in medical education can enhance students’ understanding, retention, and real-time application of medical knowledge [29], it also introduces several challenges. One significant concern is how educators will evaluate students’ work in the era of chatbots. In our cohort, some students were concerned that using ChatGPT might violate academic and university policies, while others felt comfortable utilizing it to generate content for assignments.

These advanced AI tools can generate complete essays with references that may bypass plagiarism detectors [30, 31]. Additionally, some chatbots can effectively answer multiple-choice questions [32]. This raises the challenge of distinguishing between authentic student effort and AI-generated content, which could lead to issues with evaluation, constructive feedback, and assessment. This is particularly concerning for older professors who may not be familiar with technology designed to detect AI-generated work.

Over two-thirds of our participants expressed concerns about the potential misuse of ChatGPT in medical education, underscoring the importance of establishing clear guidelines within medical schools for the use of AI tools like ChatGPT. Universities and medical schools must set standards for integrating chatbots and AI-assisted tools in academic settings. In academic research, some publishers have prohibited the use of chatbots in writing journal articles, while others allow their use for specific tasks, such as language proofreading, but not for analysis and interpretation [33]. Technology has also been developed to detect AI-assisted writing, often through specialized software or websites that can perform this function [34]. It is crucial for academics to familiarize themselves with these tools, which should be made widely accessible to the academic community.

A limited number of students in our study expressed concerns about becoming dependent on ChatGPT, potentially hindering the development of critical thinking skills. Similarly, few participants were worried about a lack of originality in university assignments and academic responsibilities resulting from its use. However, we believe this concern is significant, given how easily information can now be accessed and scientific content created. Chatbots like ChatGPT further streamline this process by quickly answering questions and providing relevant data on a wide range of topics, emphasizing the need for responsible usage and critical engagement.

Students could progressively become reliant on this technology, limiting their ability to search and utilize other resources, and potentially leading to emotional difficulties and challenges in learning and living without it. Interestingly, research suggests that interactions with chatbots can evoke emotional responses, which may influence real-life interpersonal communication and relationships [35]. Although a recent study found that this dependence was somewhat limited and not predictive of subsequent anxiety or depression, the authors concluded that it was a growing trend [36]. Further research is needed to evaluate both psychological and educational dependence on chatbots among different groups, particularly university students.

Our participants expressed mixed concerns regarding ChatGPT’s accuracy, particularly when it comes to generating information on complex medical topics. Some participants viewed ChatGPT as a reliable source of information, while others questioned the accuracy of its output. Another issue related to dependence on chatbots is the potential for “automation bias.” This bias occurs when individuals become overly reliant on AI tools, trusting them without providing new input or oversight. This leads to the assumption that the AI is always correct, often disregarding information that doesn’t align with its outputs [37]. Such bias poses significant challenges in effectively implementing AI-driven technologies.

Healthcare providers, including physicians, are increasingly utilizing AI algorithms for clinical diagnosis, treatment prescription, and identifying features in complex medical imaging, particularly in challenging cases [38]. Dependence on chatbots can exacerbate this risk of bias, especially among younger generations. Without proper human oversight, responsible design, and operation, AI applications carry the risk of generating and disseminating misinformation or harmful, inaccurate content on an unprecedented scale [39].

## Conclusions and recommendations

Our study shows that medical students exhibit a strong level of knowledge, interest, and positive attitudes towards using ChatGPT and similar chatbots in their academic work, with this trend being particularly prominent among younger students. However, there were concerns about the reliability of the information provided by these tools, the potential for misuse in medical education, and the importance of establishing clear guidelines on their use. Additionally, concerns were voiced regarding the risk of dependency on ChatGPT, which could hinder the development of critical thinking skills.

The use of these AI tools should be guided by ethical principles and guidelines that are developed and regularly updated by academic institutions to keep pace with technological advancements. Educators must be equipped with the necessary knowledge and tools to evaluate and educate students effectively in the era of AI. Future research should focus on examining the potential for dependency and emotional attachment to AI tools, particularly among younger generations, and the broader implications for their educational and personal development.

## Electronic supplementary material

Below is the link to the electronic supplementary material.

Supplementary Material 1

## Data Availability

The datasets used and/or analyzed during the current study are available from the corresponding author on reasonable request.

## Abbreviations

AI: Artificial Intelligence
GPT: Generative Pretrained Transformer
LLM: Large Language Models
