# Supplementary material for: Medical students and ChatGPT: analyzing attitudes, practices, and academic perceptions
Source: BMC Med Educ. 2025 Feb 5;25:187. doi: 10.1186/s12909-025-06731-9 (PMC11800517; doi:10.1186/s12909-025-06731-9)
Supplement: Supplementary file 1 — Supplementary Material 1 [file 12909_2025_6731_MOESM1_ESM.docx]

**Age:**

**Sex**

1. Male
2. Female

**Governorate**

1. Cairo
2. Alexandria
3. Suez
4. Beni-Suef

**University**

1. Public
2. Private

**Have you heard of ChatGPT before this study?**

1. Yes
2. No

**Have you used ChatGPT before this study?**

1. Yes
2. No

|  | **Not helpful at all** | **Not helpful** | **Neutral** | **Helpful** | **Extremely helpful** |
| --- | --- | --- | --- | --- | --- |
| **Perception** |  |  |  |  |  |
| 1.How helpful do you think ChatGPT could be in understanding medical concepts? |  |  |  |  |  |
| 2. How likely do you think it is that ChatGPT could provide accurate medical information? |  |  |  |  |  |
| 3. ChatGPT could be a helpful tool for summarizing research papers |  |  |  |  |  |

|  | **Strongly disagree** | **Disagree** | **Neutral** | **Agree** | **Strongly agree** |
| --- | --- | --- | --- | --- | --- |
| **Attitude** |  |  |  |  |  |
| 1.I am concerned about the reliability of the information provided by ChatGPT |  |  |  |  |  |
| 2. I am afraid of relying too much on ChatGPT and not developing my critical thinking skills |  |  |  |  |  |
| 3. I am concerned about the potential security risks of using ChatGPT |  |  |  |  |  |
| 4. I am afraid of becoming too dependent on technology like ChatGPT |  |  |  |  |  |
| 5. I am afraid that using ChatGPT would result in a lack of originality in my university assignments and duties |  |  |  |  |  |
| 6. I am afraid that the use of the ChatGPT would be a violation of academic and university policies |  |  |  |  |  |
| 7. I am concerned about the potential privacy risks that might be associated with using ChatGPT |  |  |  |  |  |
| 8. I am enthusiastic about using technology such as ChatGPT for learning and research. |  |  |  |  |  |
| 9. I believe technology such as ChatGPT is an important tool for academic success. |  |  |  |  |  |
| 10. I think that technology like ChatGPT is attractive and fun to use. |  |  |  |  |  |
| 11. I am always keen to learn about new technologies like ChatGPT. |  |  |  |  |  |
| 12. I trust the opinions of my friends or colleagues about using ChatGPT. |  |  |  |  |  |
| 13. I consider using ChatGPT for help with my studies |  |  |  |  |  |
| 14. I am comfortable with using ChatGPT to generate content for assignments |  |  |  |  |  |
| 15. I am concerned about the potential for misuse of ChatGPT in medical education |  |  |  |  |  |
| 16. I am concerned with the accuracy of information ChatGPT might generate on complex medical topics |  |  |  |  |  |
| 17. I think ChatGPT could be a reliable source for preparing medical exams |  |  |  |  |  |
| 18. It is important for medical schools to establish clear guidelines on the use of AI tools like ChatGPT |  |  |  |  |  |
| **Practice** |  |  |  |  |  |
| 1. ChatGPT helps me to save time when searching for information. |  |  |  |  |  |
| 2. For me, ChatGPT is a reliable source of accurate information. |  |  |  |  |  |
| 3. I recommend ChatGPT to my colleagues to facilitate their academic duties. |  |  |  |  |  |
| 4. ChatGPT is more useful than other sources of information that I have used previously. |  |  |  |  |  |
| 5. I have used tools or techniques similar to ChatGPT in the past. |  |  |  |  |  |
| 6. I spontaneously find myself using ChatGPT when I need information for my university assignments and duties. |  |  |  |  |  |
| 7. I often use ChatGPT as a source of information in my university assignments and duties. |  |  |  |  |  |
| 8. I think that relying on technology like ChatGPT can disrupt my critical thinking skills. |  |  |  |  |  |
| 9. I appreciate the accuracy and reliability of the information provided by ChatGPT. |  |  |  |  |  |
| 10. I believe that using ChatGPT can save time and effort in my university assignments and duties. |  |  |  |  |  |
| 11. It does not take a long time to learn how to use ChatGPT. |  |  |  |  |  |
| 12. ChatGPT does not require extensive technical knowledge. |  |  |  |  |  |
| 13. I am interested in using more sophisticated AI tools to personalize my learning experience |  |  |  |  |  |
